# Supplementary material for: Polymyxin Resistance in Salmonella: Exploring Mutations and Genetic Determinants of Non-Human Isolates
Source: Antibiotics (Basel). 2024 Jan 23;13(2):110. doi: 10.3390/antibiotics13020110 (PMC10885896; doi:10.3390/antibiotics13020110)
Supplement: Supplementary file 1 [file antibiotics-13-00110-s001.zip › SuplemtableS1.pdf]

Supplementary Table S1: *Salmonella* serotypes received in the period 2016-2021

**Serotypes**	**2016**	**2017**	**2018**	**2019**	**2020**	**2021**	**Total**
Abaetuba	1	1	3	9		2	16
Abony					1		1
Adelaide			3				3
Agona	1		2	2		13	18
Alachua			3				3
Albany			1				1
Anatum	2	1		2		8	13
Braenderup	10	0	11	3			24
Brandenburg	1	11					12
Bredeney	1						1
Carrau						4	4
Cerro	11		6	3		1	21
Choleraesuis			1	1			2
Coeln				1		1	2
Corvallis	1			1	2	1	5
Cubana			1		1	2	4
Derby			2	2			4
Dublin	3			1		1	5
Enteritidis	47	9	6		2	39	103
Fresno						3	3
Gallinarum		3		2	3	3	11
Glostrup		1			2		3
Grumpensis				1			1
Hadar			2				2
Havana	2						2
Heidelberg	11	27	92	6	18	15	169
Infantis	11	2	4	1	1	5	24
Inganda			3	1			4
Javiana	15	1	4	2			22
Kedougou					1		1
Kentucky						1	1
Livingstone	1		1			1	3
Madelia				3			3
Mbandaka	4		26	21	13	26	90
Meleagridis			2				2
Miami	11	1	1		1	1	15
Minnesota		1	14	6	11	1	33
Molade	3		1				4
Morehead					4	1	5
Muenchen				6	6	6	18
Muenster	24		2				26
Newport		1	1	1	14	4	21

Ohio		1				38	39
Oranienburg	1		3	1		1	6
Orion	3	2		1			6
Oslo				1			1
Ouakam			1				1
Panama	2	1	3	1	1	1	9
Poona				4	9	1	14
Potsdam	1						1
Pullorum		1			1	3	5
Reading				1			1
Rissen	2		6	5	3	1	17
Rubislaw	1		1	1		1	4
S. enterica subsp houtenae O43				1			1
S. enterica subsp. diarizonae (IIIb)	1						1
S. enterica subsp. enterica 4,5,12:-:1,2			1			1	2
S. enterica subsp. enterica 6,7:z10:-						1	1
S. enterica subsp. enterica 3,19:-:-						3	3
S. enterica subsp. enterica 4, 5, 12: eh :-			1				1
S. enterica subsp. enterica 4,12:d:-			2		2		4
S. enterica subsp. enterica 4,5,12:i:-	44	5	1	1	7	12	70
S. enterica subsp. enterica cepa rugosa		4	17	2	3	3	29
S. enterica subsp. houtenae	2						2
S. enterica subsp. houtenae 16:z4,z23:-				1			1
S. enterica subsp. houtenae 43:gz51:-		1					1
S. S. enterica subsp. houtenae 43:g,z51:-		1					1
S.enterica subsp. enterica 4,12: r: -			1				1
S.enterica subsp. enterica 6,8:eh:-	1						1
S.enterica subsp. salamae 42:r:-	1			1			2
Saintpaul	1	3	5	8	1		18
Salmonella enterica subsp. diarizonae					2		2
Salmonella enterica subsp. enterica			6		1	13	20
Salmonella enterica subsp. enterica 6,8:r:-				1			1
Salmonella enterica subsp. salamae					3	4	7
Salmonella enterica subsp. salamae O42				1			1
Sandiego	1	7	1	4	1		14
Saphra				3	2	1	6
Schwarzengrund	5		15		4	1	25
Senftenberg	2	1	7		1	7	18
Soerenga						1	1
Tennesse	1		4	2		7	14
Typhimurium	11	2	13	5	10	39	80
Winslow						1	1

Worthington	1	1				6	8
Yoruba	2		1		1	1	5
Zega			3				3
Montevideo						1	1
**Total**	**243**	**89**	**284**	**120**	**132**	**288**	**1156**
